# Effects of Lumbar Stabilization on Scapular Muscle Activity, Activation Onset Time, and Kinematics in Individuals with Scapular Dyskinesis

**DOI:** 10.5114/jhk/186972

**Published:** 2024-07-17

**Authors:** Sumarttra Sungkue, Prasert Sakulsriprasert, Mantana Vongsirinavarat, Nalut Utsahachant, Mark P. Jensen

**Affiliations:** 1 Faculty of Physical Therapy, Mahidol University, Nakhon Pathom, Thailand.; 2 Department of Rehabilitation Medicine, University of Washington, Washington, United States.

**Keywords:** scapula, electromyography, muscle recruitment timing, abdominal muscles

## Abstract

This study aimed to investigate the effects of lumbar stabilization on muscle activity, muscle onset time, and scapular kinematics in individuals with scapular dyskinesis. Fourteen participants with scapular dyskinesis were recruited. Scapular muscle activity and activation onset time were measured by electromyography (EMG), and scapular upward rotation was measured by two-dimensional (2-D) Kinovia software, under two conditions: with and without abdominal bracing. There was a significant increase in the activity of the serratus anterior, middle trapezius, and inferior trapezius muscles between the conditions (p < 0.001, p = 0.045, and p < 0.001, respectively). During abdominal bracing, the activation onset time of the serratus anterior and lower trapezius was noticeably shorter (p = 0.041 and p = 0.011, respectively). Scapular upward rotation at 30°, 60°, 90°, and 120° of shoulder abduction was significantly greater (p = 0.027, p = 0.003, p = 0.003, and p = 0.030, respectively). Increased scapular muscle activation, early activation onset time, and increased scapular upward rotation were also noted. These changes have an impact on the scapulohumeral rhythm.

## Introduction

Scapular dyskinesis can be found in 50%–68% of healthy individuals (Hannah et al., 2017; Myers et al., 2013). It is commonly associated with several pathologies of the shoulder, especially subacromial impingement syndrome (SIS) (Kibler et al., 2013). Individuals with scapular dyskinesis have altered relationships and balance in the activity of different muscles, specifically less activity in both the serratus anterior and lower trapezius muscles and more activity in the upper trapezius muscle (Huang et al., 2015). Extended overactivity of the upper trapezius muscle may result in fatigability of the muscles, which in turn alter scapular kinematics (Huang et al., 2022). Although muscle activity differences have been shown between people with and without scapular dyskinesis, differences in recruitment times of these muscles have not always been reported (Struyf et al., 2014). The scapular muscle imbalance can cause alterations in the scapular muscles force-couple and is related to the lack of scapular upward rotation (Seitz et al., 2015). Generally, the serratus anterior provides upward rotation, a posterior tilt, and external rotation of the scapula (Phadke et al., 2009). However, individuals with scapular dyskinesis have a risk of impingement by insufficient scapular upward rotation and the posterior tilt (Kibler, 1998; Voight and Thomson, 2000), especially during arm lowering (Huang et al., 2015). More specifically, scapular dyskinesis has been associated with a 43% increased risk of shoulder pain in overhead athletes (Hickey et al., 2018). While shoulder pain in overhead athletes is frequently complicated, scapular dyskinesis treatment and early diagnosis result in improved patient outcomes (Saini et al., 2020).

To improve scapular dyskinesis, rehabilitation aims to change scapular kinematics to improve both the scapular position and motion. The effects of improving scapular kinematics in individuals with scapular dyskinesis are not known (Nodehi Moghadam et al., 2020). Altered scapular kinematics have been linked to muscle activity not only related to the control of the scapula, but also to muscle activity related to trunk control (Burkhart et al., 2003).

Based on the theory of anatomical linkage, trunk muscles should be anatomically and functionally related to the scapular muscles, so that the contraction of abdominal muscles could potentially facilitate scapular muscle activity and generate efficient dynamic movements (Kibler, 1995; Myers, 2014). Consistently with this hypothesis, several studies have shown that abdominal contraction can increase serratus anterior muscle activity during the arm forward punch and arm elevation (Bielec et al., 2013; Kaur et al., 2020; Scott et al., 2018; Gunaydin, 2021). Moreover, the abdominal contraction has been shown to decrease the latencies of the serratus anterior, lower trapezius, and shoulder muscles during arm elevation, which reflects the improvement of joint stability (Scott et al., 2018). Interestingly, the facilitation of abdominal muscles to increase muscle activation of the serratus anterior may affect scapular kinematics by decreasing scapular internal rotation (Kim et al., 2017). Although these preliminary findings suggest that abdominal muscle contraction could potentially improve periscapular muscle function in normal scapular mobility, no study has evaluated the effects of abdominal muscle contraction in individuals with scapular dyskinesis, which have also been linked to altered kinematics of the shoulder joint.

Given these considerations, this study aimed to evaluate the impact of lumbar stabilization on periscapular muscles in individuals with scapular dyskinesis. We hypothesized that there would be more activity in the periscapular muscles with versus without lumbar stabilization across different angles during both the raising and lowering phases of shoulder abduction. In addition, we anticipated that lumbar stabilization would reduce muscle activation onset time for periscapular muscles, but would increase the scapular upward rotation angle.

## Methods

### Participants

This study was approved ethically by the Mahidol University-Central Institutional Review Board (MU-CIRB) (approval code: COA No. 2021/188.1409; approval date: 14 September 2021). The sample size was calculated using data from a pilot study of five participants. The serratus anterior, which has a direct link to the abdominal muscle (Myers, 2014), is the primary muscle responsible for scapular upward rotation during arm elevation (Neumann, 2017). The result of the pilot study was regarded as the %maximum voluntary isometric contraction (MVIC) of the serratus anterior during shoulder abduction, and shoulder abduction with abdominal bracing in individuals with scapular dyskinesis. The effect size along with mean and standard deviation of %MVIC of the serratus anterior in shoulder abduction and shoulder abduction with abdominal bracing showed values of 1.08, 25.95 ± 20.10, and 55.56 ± 31.24, respectively. For the sample size calculation, software G*power from the Wilcoxon signed-rank test was set to alpha of 0.05. The results indicated that a sample size of 12 would be needed to detect a significant effect. Participants were included if they met the following criteria: an individual without any shoulder complaint, aged between 20 and 50 years (Buonocore et al., 2012; Deschenes, 2004), and a body mass index between 18.5 and 26.9 kg/m^2^ (Gupta et al., 2013). They also must have been diagnosed with obvious scapular dyskinesis type I or II or mixed type I and II on at least one side of one of the shoulders. Exclusion criteria included: a history of fracture of an upper extremity or the spine, history of shoulder injury or shoulder surgery within the past year, pain in or near the shoulder, the scapula or the spine, pain associated with cervical radiculopathy, spinal deformity, neurological diseases, and thoracic kyphosis of more than 45°. Assuming a dropout rate of 15%, a total of 14 participants were recruited for the study.

### Measures

Muscle activation and activation onset time were measured by surface electromyogram (sEMG) (EMG and sensor system, TeleMyo DTS Telemetry, Noraxon USA, Inc, Arizona, USA). The impedance was set as not more than 10 Kiloohm (KΩ) and not having more than 20 mm of interelectrode distance. The electrode placement and interelectrode distance were determined in accordance with the guidelines provided by SENIAM (Surface ElectroMyoGraphy for the Non-Invasive Assessment of Muscles) (Hermens et al., 2000). The sEMG electrodes were placed on the target muscles as follows: for the upper trapezius at the halfway between the acromion and C7, for the middle trapezius at the middle between the root of the spine of the scapula and T3, for the lower trapezius on the line between the spine of the scapula and T7, for the serratus anterior at the anterior to the latissimus dorsi and posterior to the pectoralis major, and for the external abdominal oblique at the anterior to serratus anterior and lateral to linea semilunaris. In addition, electrodes were placed on the middle part of the deltoid of the shoulder with scapular dyskinesis, midway between the deltoid tuberosity and the acromion.

A standard universal goniometer was used to measure the angle of shoulder abduction (Kolber and Hanney, 2012), then the angles were marked with wedge-colored stripes on the wall as the reference line. Participants could raise their arms, and we took pictures in the same position using an HDR-CX210, Sony Corporation with a sampling rate of 50–60 Hz. Scapular kinematics was measured from the picture using 2-D motion analysis Kinovea software 0.8.27. The 2-D Kinovea software is a reliable and valid tool that accurately measures distances up to 5 m from the object and at an angular perspective of 90°; the details of the methodology can be found in a previous study (Puig-Diví et al., 2019).

### Design and Procedures

All participants provided written, informed consent before study participation. The scapular dyskinesis test was used to define normal, subtle, and obvious scapular dyskinesis (McClure et al., 2009). Participants were asked to perform five repetitions of bilateral, active, weighted shoulder flexion, and bilateral, active, weighted shoulder abduction at a speed matching the beat of a metronome (60^°^/s). Tests were performed with participants holding dumbbells based on their body weight (1.4 kg for body weight < 68.1 kg and 2.3 kg for body weight > 68.1 kg). We categorized the dyskinesis type using Kibler’s classification (Kibler et al., 2002). There were two physical therapists: one with over ten years of clinical expertise managing musculoskeletal disorders, and another with over five years of clinical experience. We also provided didactic presentations and the assessor was provided with a review of the information about the standardized operational definitions of scapular dyskinesis types and the scapular dyskinesis test (SDT). A hands-on practice session was then conducted as the assessor was practicing to rate the test movements of the SDT. The determination of whether ten participants presented normal motion, subtle dyskinesis or obvious scapular dyskinesis with the predominant scapular movement pattern (McClure et al., 2009) was undertaken. The practice sessions were videotaped and reviewed with the assessor. After data collection, each participant's video was then independently rated. As a result, the Kappa coefficient of agreement for classification, the type, and severity of scapular dyskinesia was 0.86, indicating excellent validity.

After the evaluation of scapular dyskinesis, data regarding kinematics and sEMG were collected. Maximal voluntary isometric contraction (MVIC) was tested and used to normalize the sEMG data during the task. For the upper trapezius muscle, MVIC was measured during resisted shoulder elevation and lateral neck flexion (Kendall, 2005). For the middle trapezius muscle, participants lay prone with the arm abducted at 90°. Resistance was applied against horizontal abduction (Hislop et al., 2013). Participants were in a prone position with 130° of arm abduction in line. Resistance was applied against further elevation (Hislop et al., 2013). For measurement of MVIC of the serratus anterior muscle, participants sat with the shoulder elevation at 125° flexion with the scapula fully upwardly rotated. Resistance was applied against shoulder flexion and against the direction of scapular upward rotation (Hislop et al., 2013). For measurement of MVIC of the external abdominal oblique muscle, participants sat with their knees flexed on the table. Participants performed an oblique sit-up attempt to move the shoulder toward the opposite knee. Resistance was applied against flexion and rotation (Kaur et al., 2020). Each of the three MVIC trials against manual resistance of the examiner lasted 5 s, with 60-s rest intervals between trials and 2-min rest intervals between different muscles for preventing fatigue (de Araújo et al., 2018).

Participants were asked to elevate their arms from the resting position, using the thumb-up position, to the end range of shoulder abduction in the coronal plane, then down to the resting position at a speed matched with a metronome (6 s). All participants performed five repetitions of shoulder abduction with 5-s rest intervals in between. After 5 min of recovery, the examiner randomly measured shoulder abduction in the frontal plane at rest, at the angles of 30°, 60°, 90°, and 120°. Afterwards, the examiner placed four markers on the spinous process of C7 and T7 at the resting position, and two markers on the medial end of the root of the spine and the inferior angle of the scapula, then the examiner captured the picture. For other angles, participants were asked to perform shoulder abduction from resting until reaching the reference line at each angle. Meanwhile, the examiner changed two markers on the medial end of the root of the spine and the inferior angle of the scapula along with the scapular movement that was followed by changing the degree of shoulder abduction. Then the participant turned to a resting position. After that, participants performed active shoulder abduction at each angle again, while the examiner captured the picture.

For the lumbar stabilization condition, shoulder abduction was performed with abdominal bracing for activating global muscles, especially the external abdominal oblique muscle (McGill et al., 2019). Participants were instructed to activate the abdominal muscle until they could increase the percentages of MVIC of the external abdominal oblique to a moderate activation level that required not less than 20% MVIC (Vezina and Hubley-Kozey, 2000) throughout shoulder abduction with EMG sound feedback, without moving their trunk or hollowing the lower abdomen. Five repetitions with 2-min rest intervals in between were conducted. EMG data for all the ranges were defined by a trigger mark and corresponded with a measuring device that was placed on the wall at the level of the shoulder joint of each participant. After 5 min of rest, shoulder abduction at rest and at the angles of 30°, 60°, 90°, and 120° was measured. Participants performed abdominal bracing before elevating the shoulder and maintained the action when the examiner placed the two markers. They were provided with a 2-min rest interval between subsequent repetitions. Participants were then asked to perform abdominal bracing during active shoulder abduction at each angle again, while the examiner took a photograph of the participant. Scapular upward rotation was measured using 2-D Kinovea software. The intra-tester reliability (ICC3,1) ranged from 0.972 to 0.991, indicating excellent reliability.

Raw EMG data were subsequently bandpass filtered (between 20 and 450 Hz), then rectified, and finally smoothed using RMS calculation with a 50-ms sliding window (Kaur et al., 2020). Resting EMG activity was considered as baseline. The EMG data for each muscle were averaged for each phase of the middle three trials. A phase was defined by a trigger mark and corresponded with a measuring device placed on the wall at the level of the shoulder joint of each participant. Muscle activity of the upper trapezius, middle trapezius, lower trapezius, serratus anterior, and external abdominal oblique muscles was recorded at the ranges of 0° to 30°, 30° to 60°, 60° to 90°, 90° to 120°, and >120° in the raising and lowering phases of arm elevation in the coronal plane. Muscle onset time was defined as the time when muscle activity exceeded 2 SD above the baseline of each muscle contraction lasting for 50 ms (Hodges and Bui, 1996).

The scapular upward rotation of each participant was measured using pictures taken at rest and at 30°, 60°, 90°, and 120° of shoulder abduction, both with and without lumbar stabilization. Firstly, the first line was drawn passing through the center of C7 and T7 vertically. An oblique was also drawn passing through the center of the root of the spine of the scapula and the inferior angle of the scapula markers. Then, the intersection between these two lines was the angle of scapular upward rotation (Wang et al., 1999).

### Statistical Analysis

Interobserver agreement of scapular dyskinesis was estimated using Kappa statistics. Descriptive statistics were computed to describe the study sample and included age, sex, and the BMI. Intraobserver reliability for measuring scapular upward rotation was assessed using the ICC (3,1). The study variables, including measures of scapular muscle activity, muscle onset time, and the angle of scapular upward rotation in shoulder abduction with and without lumbar stabilization were tested for normality using the Shapiro-Wilk test. The variables in this study were normally distributed, therefore, two-way repeated measures ANOVA was used to examine the differences in EMG data for the ranges of 0° to 30°, 30° to 60°, 60° to 90°, 90° to 120°, and >120° in both raising and lowering phases, and those in kinematic data at rest, 30°, 60°, 90°, and 120° of shoulder abduction between conditions of with and without lumbar stabilization. When differences between pairs were found, the post-hoc pairwise analysis was performed using the least significant difference (LSD) test. The alpha level used to determine statistical significance for the primary analysis was set at p<0.05. All analyses were conducted using SPSS version 23.0 (IBM Corp, Armonk, NY, USA).

## Results

**Figure 1:**
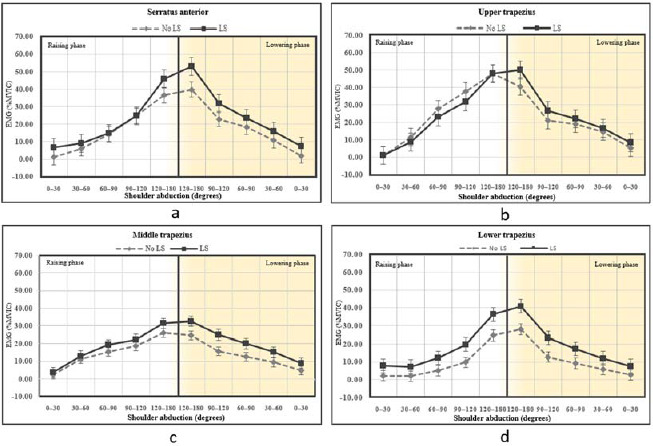
Means of muscle activation for both raising and lowering shoulder abduction without and with lumbar stabilization. EMG, electromyogram; MVIC, maximal voluntary isometric contraction; LS, lumbar stabilization. * Significant difference between no LS and with LS (p < 0.05)

A total of 14 individuals with scapular dyskinesis participated in the study. The demographic data are reported in Table 1. The EMG data of both conditions are reported in Table 2 and Figure 1. No interaction between angles and conditions was found in any muscle such as the serratus anterior, upper trapezius, middle trapezius and lower trapezius muscles (F(1.9)=2.44, p=0.064, (ES)=0.086, F(1.9)=2.35, p=0.098, ES=0.083, F(1.9)=1.34, p=0.270, ES=0.049 and F(1.9)=1.93, p=0.127, ES= 0.069, respectively). There was a main effect for the phase under both conditions on the serratus anterior, upper trapezius, middle trapezius, and lower trapezius muscles (F(1.9)=127.57, p<0.001, ES=0.831, F(1.9)=106.73, p<0.001, ES=0.804, F(1.9)=56.24, p<0.001, ES=0.684, and F(1.9)=102.59, p<0.001, ES=0.798, respectively).

There was also a main effect of conditions on the serratus anterior, middle trapezius, and lower trapezius muscles (F(1.26 =16.62, p<0.001, ES=0.390, F(1.26)= 4.45, p=0.045, ES=0.146, and F(1.26)=21.27, p<0.001, ES=0.450, respectively). The muscle activation of the upper trapezius was not significantly different between conditions (F(1.26)=0.49, p=0.489, ES=0.019).

**Table 1:** Participants’ demographic data.

Characteristics	n = 14
Gender (male/female), n (%)	4 (29%) / 10 (71%)
Age (years), mean ± SD	31.00 ± 8.62
Body Height (cm), mean ± SD	160.14 ± 7.04
Body Mass (kg), mean ± SD	54.88 ± 7.67
Body Mass Index (kg/m^2^), mean ± SD	21.34 ± 1.96

*SD: standard deviation*

**Table 2:** Means of muscle activation for both raising and lowering shoulder abduction without and with lumbar stabilization.

Condition	Raising phase mean % MVICs (95% CIs)					Lowering phase mean % MVICs (95% CIs)					*F* for condition effect	*F* for phase effect	*F* for condition x phase effect interaction
	0°–30°	30°–60°	60°–90°	90°–120°	>120°	>120°	90°–120°	60°–90°	30°–60°	0°–30°			
Serratus anterior													
No LS	1.14_a_ (-1.06, 3.34)	6.61_b_ (4.05, 8.16)	14.28_c_ (11.51, 17.06)	24.72_d_ (20.44, 29.00)	36.43_e_ (31.37, 41.50)	39.77_e_ (33.29, 46.25)	22.98_d_ (16.99, 28.98)	18.53_c_ (14.13, 22.92)	10.77_f_ (7.81, 13.73)	1.90_a_ (0.41, 3.40)	16.62* (*p* < 0.001)	127.57* (*p* < 0.001)	2.44 (*p* = 0.064)
LS	6.60_x,a_ (4.40, 8.80)	9.14_x,b_ (7.09, 11.19)	14.79_c_ (12.01, 17.56)	24.77_d_ (20.49, 29.05)	45.96_x,f_ (40.90, 51.02)	53.07_x,g_ (46.59, 59.55)	31.72_x,d(_ (25.73, 37.71)	23.39e (19.00, 27.79)	15.87_x,c_ (12.91, 18.82)	7.52_x,b_ (6.02, 9.01)			
Upper trapezius													
No LS	0.98_a_ (0.53, 1.42)	11.62_b_ (8.12, 15.13)	27.68_c_ (21.56, 33.80)	37.77_d_ (30.28, 45.26)	47.93_e_ (43.22, 52.63)	40.50_d_ (33.53, 47.47)	21.23_c_ (17.27, 25.20)	19.05_f_ (15.71, 22.39)	14.68_b_ (11.52, 17.83)	5.39_g_ (2.76, 8.02)	0.49 (*p* = 0.489)	106.73* (*p* < 0.001)	2.35 (*p* = 0.098)
LS	1.32_a_ (0.88, 1.77)	8.65_b_ (5.14, 12.15)	23.30_cde_ (17.18, 29.42)	31.80_f_ (24.32, 39.29)	48.04_g_ (43.33, 52.74)	50.15_g_ (43.18, 57.12)	26.57_cf_ (22.60, 30.53)	21.99_d_ (18.65, 25.34)	16.77_e_ (13.62, 19.93)	8.34_b_ (5.71, 10.97)			
Middle trapezius													
No LS	2.28_a_ (0.93, 3.64)	11.23_b_ (7.57, 14.89)	15.29_de_ (10.65, 19.93)	18.62_fg_ (13.90, 23.33)	25.84_h_ (19.64, 32.05)	24.56_fh_ (16.44, 32.68)	15.75_dg_ (11.01, 20.48)	12.51_be_ (8.24, 16.77)	9.37_c_ (6.02, 12.71)	4.64_i_ (2.48, 6.80)	4.45* (*p* = 0.045)	56.24* (*p* < 0.001)	1.34 (*p* = 0.270)
LS	3.65_a_ (2.30, 5.01)	12.96_bc_ (9.30, 16.62)	19.28_d_ (14.64, 23.92)	22.27_ef_ (17.55, 26.99)	31.34_g_ (25.14, 37.55)	32.55_g_ (24.43, 40.67)	24.89_x,e_ (20.16, 29.63)	20.02_x,df_ (15.76, 24.29)	15.32_x,b_ (11.97, 18.66)	8.98_x,c_ (6.81, 11.14)			
Lower trapezius													
No LS	2.23_a_ (-0.26, 4.72)	2.04_a_ (0.28, 3.81)	5.15_bc_ (1.73, 8.57)	9.71_def_ (4.75, 14.68)	24.72_g_ (18.97, 30.46)	28.04_g_ (22.65, 33.44)	12.64_d_ (8.35, 16.93)	9.17_e_ (5.29, 13.04)	5.84_bf_ (3.31, 8.38)	2.71_ac_ (1.05, 4.36)	21.27* (*p* < 0.001)	102.59* (*p* < 0.001)	1.93 (*p* = 0.127)
LS	7.71_x,a_ (5.23, 10.20)	7.27_x,a_ (5.51, 9.04)	12.23_x,b_ (8.81, 15.65)	19.76_x,cd_ (14.79, 24.72)	36.36_x,e_ (30.61, 42.11)	40.9_x,f_ (35.54, 46.32)	23.37_x,c_ (19.08, 27.66)	17.24_x,d_ (13.36, 21.11)	12.0_x,b_ (9.52, 14.59)	7.61_x,a_ (5.96, 9.26)			

MVIC, maximal voluntary isometric contraction; IC, confidence interval; LS, lumbar stabilization; * indicates means that are significantly different between conditions (p < 0.05); x subscripts indicate means that are significantly different between conditions (condition effect); a, b, c, d, e, f, g, h, and i subscripts indicate means that are significantly different within the group (phase effect)

Table 2 summarizes the differences in scapular muscle activation for all the ranges in both the raising and lowering phases between the two conditions. There were significant differences in muscle activity between conditions in the serratus anterior in almost every phase in the raising and lowering phases. The EMG activity of the middle trapezius was also significantly different between conditions, especially in arm lowering. Significant differences were also found in lower trapezius muscle activity between without and with abdominal bracing for all the ranges of arm raising and lowering. Table 3 shows that muscle activation onset time was different between conditions for both the lower trapezius (p=0.041, ES=0.81) and the serratus anterior (p=0.011, ES=1.05). There was no significant difference between conditions in muscle activation onset time of the middle deltoid, upper trapezius, and middle trapezius muscles (p=0.887, ES=0, p=0.785, ES=0.11, and p=0.640, ES=0.18, respectively).

**Table 3:** Means of muscle activation onset time (in seconds) at raising shoulder abduction without and with lumbar stabilization.

Muscle (Mean ± SD)	Condition		*p*-value	ES
	No LS	With LS		
Middle deltoid	0.34 ± 0.10	0.34 ± 0.12	0.887	0
Upper trapezius	0.51 ± 0.16	0.53 ± 0.19	0.785	0.11
Middle trapezius	0.47 ± 0.14	0.50 ± 0.19	0.640	0.18
Lower trapezius	1.06 ± 0.52	0.66 ± 0.47	0.041*	0.81
Serratus anterior	0.65 ± 0.20	0.44 ± 0.20	0.011*	1.05

SD, standard deviation; LS, lumbar stabilization; ES, Cohen’s d effect size * Significant difference between no LS and with LS (p < 0.05)

Scapular kinematics presented with angles of upward rotation during raising shoulder abduction with and without lumbar stabilization is shown in Table 4. There were significant increases between conditions at 30°, 60°, 90°, and 120° (p=0.027, ES=0.88, p=0.003, ES=1.26, p=0.003, ES=1.22, and p=0.030, ES=0.87, respectively).

**Table 4:** Means of the scapular upward rotation angle during raising shoulder abduction without and with lumbar stabilization.

Raising phase in angle (Mean ± SD)	Condition		*p*-value	ES
	No LS	With LS		
Rest	2.79 ± 3.36	5.14 ± 4.31	0.119	0.61
30°	5.29 ± 3.10	8.36 ± 3.82	0.027*	0.88
60°	11.36 ± 3.34	15.29 ± 2.89	0.003*	1.26
90°	20.00 ± 3.14	23.79 ± 3.07	0.003*	1.22
120°	30.00 ± 2.69	32.93 ± 3.93	0.030*	0.87

SD, standard deviation; LS, lumbar stabilization; ES, Cohen’s d effect size * Significant difference between no LS and with LS (p < 0.05)

## Discussion

This study investigated the EMG muscle activity, activation onset time of periscapular muscles, and scapular kinematic variables among individuals with dyskinesis during shoulder abduction. There were significant differences in the serratus anterior, middle trapezius, and lower trapezius activity between conditions. Deep breathing is used during abdominal bracing to increase intra-abdominal pressure, which stabilizes the lumbar spine (Tayashiki et al., 2016). Abdominal bracing has been proven to help activate internal abdominal oblique and external abdominal oblique activity because it engages the abdominal muscles without inflating or deflating the lower abdomen (Tayashiki et al., 2016). The effect of greater abdominal activity, which aims to stabilize the lumbar spine, reaches distally beyond the trunk, as seen by the increased activation of key scapular stabilizing muscles. The current study is consistent with the theory of proximal to distal control, kinetic chains, and anatomy trains. Particularly, of note are the connections of the spiral line from the external abdominal oblique to the serratus anterior (Myers, 2014), and the posterior sling from the interconnection of the thoracolumbar fascia with the external abdominal oblique, which may destabilize the scapula by increasing the activation of the lower trapezius (Murta et al., 2020). Moreover, our results showed increased EMG activity of the middle trapezius only in the lowering phase. The findings may reflect the possibility that the middle trapezius acts as a stabilizer through the eccentric contraction to control the changing position of the scapula produced by the serratus anterior and the upper trapezius (Camargo and Neumann, 2019). In addition, higher upper trapezius EMG activity following lumbar stabilization may also be explained by the serratus anterior and the upper trapezius working together to stabilize the scapula, and encourage clavicular elevation during arm elevation (Scott et al., 2018).

Compared to healthy subjects, those with subacromial impingement syndrome who exhibit scapular dyskinesis have an earlier activation onset time of the upper trapezius during raising under the unloaded condition (Phadke and Ludewig, 2013). However, no differences in muscle activation onset time of the upper trapezius and the serratus anterior for the unloaded condition have been observed, and the lower trapezius was the last to be activated (Phadke and Ludewig, 2013). At the initial movement, muscle activation onset time is often used to represent neuromuscular control around a joint system (Larsen et al., 2013). One prior study that investigated the latency in healthy individuals with and without scapular dyskinesis showed no significant difference in latency of the trapezius and the serratus anterior for the unloaded condition (Petviset et al., 2021). Our findings show that scapular muscle activation onset time in asymptomatic scapular dyskinesis appears to be the same as that found in healthy individuals. The exception was the lower trapezius which appeared to have delayed activation considering the onset time (approximately 1.06 s). However, the activation onset time of the serratus anterior and lower trapezius muscles appeared to be earlier during performing lumbar stabilization. Earlier muscle onset time clusters may reflect the improvement in shoulder dynamic stability during movement (Scott et al., 2018). We speculated that early activation was associated with the anticipated consequences of movement start timings (Oshikawa et al., 2022) and verbal instruction, which was the simplest method of activating the lumbar level (Becker et al., 2022).

The results of the current study demonstrated that performing lumbar stabilization at approximately 20–40% MVIC (a low to a moderate level) caused an increase in the EMG activity of the serratus anterior and the lower trapezius at approximately <10%–40% MVIC (a minimal to a moderate level). In a prior study, the abdominal contraction was observed to be related to enhanced serratus anterior activity by around 40% MVIC with the decreased amount of scapular winging (Kim et al., 2017). During shoulder abduction and flexion, the lower trapezius and the serratus anterior are the primary upward rotators of the scapula (Neumann and Camargo, 2019). Previous studies have noted the importance of strengthening or increasing the control of the serratus anterior and the lower trapezius to reduce the likelihood of shoulder impingement (Camargo and Neumann, 2019; Kibler et al., 2013). Additionally, the mean difference of the scapular upward rotation angle in the current study was approximately 3^°^. The therapeutic significance of improving scapular upward rotation has not been determined, however, a prior study found that deviations or changes of 2°–3° are important (Michener et al., 2016). The present study investigated whether the incorporation of lumbar stabilization may alter the kinematics, activation, and onset time of the scapular muscle, which may impact the scapulohumeral rhythm. Because of its ability to optimize scapulohumeral rhythm, the scapula is an essential link in the chain (Kibler and Sciascia, 2019). To enhance performance in the training process, especially for overhead athletes who have shoulder problems, the initial step could begin with abdominal contractions to facilitate the scapula during arm elevation (Sciascia et al., 2012). Additionally, reconsideration should also be given to rehabilitation techniques that prioritize improving motor control over strength (Sciascia and Kibler, 2022).

Limitations of this study should be noted. First, our research focused on muscle activation onset time, a significant factor in determining stability during movement, but activation time and deactivation time are also required. Second, this study focused on the immediate effect of abdominal bracing during shoulder abduction in a standing position. Although this suggests the idea that additional abdominal bracing might improve the longer-term benefits of training, this possibility needs to be tested in future studies. Third, considering that we focused on the serratus anterior, the study might not be relevant for other muscles. The effect size was large, but because of the small sample size used in this pilot study, its confidence interval might be quite wide. Last, participants were adults without shoulder symptoms. Therefore, the results of the study may not be generalized to younger or older individuals. This topic needs to be studied further to be applied to various age groups, individuals with shoulder pathology, and to make comparisons with other groups, including healthy participants.

## Conclusions

This study aimed to examine the effects of lumbar stabilization on the muscle activation of the periscapular muscles. It was found that activation of the serratus anterior, middle, and lower trapezius muscles was increased. Early activation onset time of the serratus anterior and the lower trapezius, as well as increased scapular upward rotation were also noted. These changes are promising for further study regarding the rehabilitation of scapular dyskinesis.
